# Towards a New Understanding of Decision-Making by Hematopoietic Stem Cells

**DOI:** 10.3390/ijms21072362

**Published:** 2020-03-29

**Authors:** Geoffrey Brown

**Affiliations:** School of Biomedical Sciences, Institute of Clinical Sciences, University of Birmingham, Birmingham B15 2TT, UK; g.brown@bham.ac.uk; Tel.: +0121-414-4082

**Keywords:** hematopoiesis, stem cells, decision-making, cytokines, environmental niches

## Abstract

Cells within the hematopoietic stem cell compartment selectively express receptors for cytokines that have a lineage(s) specific role; they include erythropoietin, macrophage colony-stimulating factor, granulocyte colony-stimulating factor, granulocyte/macrophage colony-stimulating factor and the ligand for the fms-like tyrosine kinase 3. These hematopoietic cytokines can instruct the lineage fate of hematopoietic stem and progenitor cells in addition to ensuring the survival and proliferation of cells that belong to a particular cell lineage(s). Expression of the receptors for macrophage colony-stimulating factor and granulocyte colony-stimulating factor is positively autoregulated and the presence of the cytokine is therefore likely to enforce a lineage bias within hematopoietic stem cells that express these receptors. In addition to the above roles, macrophage colony-stimulating factor and granulocyte/macrophage colony-stimulating factor are powerful chemoattractants. The multiple roles of some hematopoietic cytokines leads us towards modelling hematopoietic stem cell decision-making whereby these cells can ‘choose’ just one lineage fate and migrate to a niche that both reinforces the fate and guarantees the survival and expansion of cells as they develop.

## 1. Introduction

Longstanding principles to the nature of hematopoietic stem cells (HSCs) are that they are strictly multipotent and able to self-renew. HSCs sustain these behaviours by residing in supportive niches in the bone marrow, as first outlined in Schofield’s stem cell niche model in 1978. Schofield also proposed that if the progeny of HSCs are unable to occupy a stem cell niche “they proliferate and mature to acquire a high probability of differentiation” [1]. The cell types that HSCs associate with has been the subject of extensive investigation and they include both nonhematopoietic cells, e.g., perivascular mesenchymal stem and endothelia cells, and hematopoietic cells, e.g., megakaryocytes, macrophages and regulatory T cells [2]. They provide the cell-bound and secreted factors that regulate various aspects of the behaviour of HSCs. For example, stem cell factor (SCF), which binds to the receptor tyrosine kinase c-Kit on HSCs, plays a role in HSC localising within the endosteal marrow region [3,4]. SCF also promotes HSC survival, proliferation and self-renewal [5,6].

The Schofield model still very much underpins hematopoiesis and the progeny of HSCs, termed hematopoietic progenitors cells (HPCs), are decision-making in terms of choosing a pathway(s) of development to follow towards a mature cell type. They are also the compartment that undergoes cell expansion which occurs to generate the large number of mature cells required from HSCs. However, recent findings contradict the notion that HSCs are a homogeneous population of pluripotent cells. This review examines the extent to which there are subpopulations of HSCs that express cytokine receptors that have a lineage(s) specific role and with a particular lineage bias as revealed by transplantation studies. New findings move the adoption of just one cell lineage to the HSC stage of development, as opposed to the level of downstream HPCs. The continuum model for hematopoiesis, proposed by Brown and colleagues, envisages HSCs choosing directly from the entire range of hematopoietic cell fates and then developing along this pathway [7,8]. Decision-making at the level of HSCs and the fact that these cells reside in a specialised niche(s) also raises an interesting question regarding the instructive role of the niches in HSCs affiliating to a developmental pathway.

## 2. The Selective Expression of Cytokine Receptors by Hematopoietic Stem Cells

The action of hematopoietic cytokines regarding cell survival and proliferation is permissive because the cytokine is a selection factor that allows a cell to survive and proliferate. From the discovery of hematopoietic cytokines, we have known that the following cytokines have permissive lineage(s) specific roles. Macrophage colony-stimulating factor (M-CSF) supports the development of macrophages and dendritic cells [9]; erythropoietin (Epo) the survival and proliferation of erythroid progenitor cells [10]; granulocyte colony-stimulating factor (G-CSF) supports granulocyte precursors [11,12]; and granulocyte/macrophage colony-stimulating factor (GM-CSF) supports the precursors of granulocytes and macrophages [13]. Thrombopoietin (Tpo) is important to megakaryocyte and platelet production [14]. Cell surface expression of the fms-like tyrosine kinase 3 (Flt3), for Flt3 ligand, delineates progenitors of lymphoid and myeloid cells from those developing towards megakaryocytes and erythrocytes [15].

**Figure 1:**
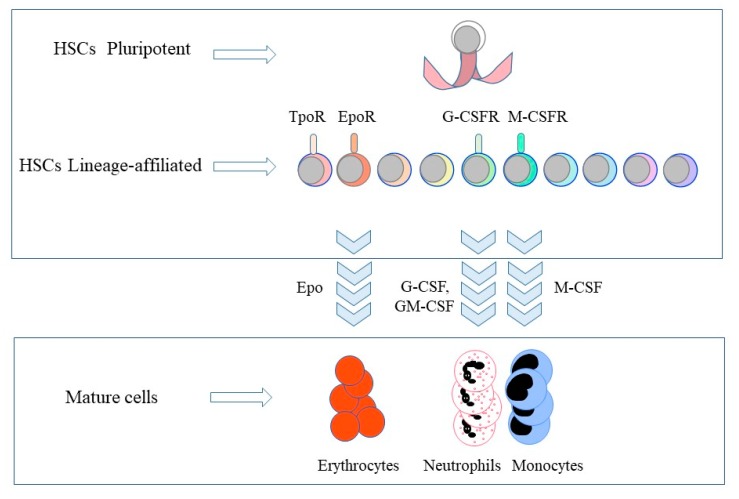
The expression of lineage-affiliated cytokine receptors by hematopoietic stem cells and the lineage instructive cytokines. Hematopietic stem cells (HSCs) express the receptors (R) for thrombopoietin (Tpo), erythropoietin (Epo), granulocyte colony-stimulating factor (G-CSF) and macrophage colony-stimulating factor (M-CSF). Epo commits HSCs and multipotent HPCs to erythropoiesis. M-CSF instructs myeloid fate in HSCs and macrophage fate in granulocyte/macrophage progenitors. G-CSF and granulocyte/macrophage colony-stimulating factor (GM-CSF) provoke the generation of neutrophils from granulocyte/macrophage progenitors. TpoR, thrombopoietin receptor; G-CSFR, granulocyte colony-stimulating factor receptor; M-CSFR, macrophage colony-stimulating factor receptor.

Murine HSCs can be FACS sorted into long-term reconstituting HSC (LT-HSC, Lineage-ve, Sca-1+ve, c-Kit+ve (LSK) CD150+ve CD48-ve CD34-ve) or short-term reconstituting (ST-HSC, LSK CD150+ve CD48-ve CD34+ve) based on their expression of CD34 [16]. Both LT-HSCs and ST-HSCs express cytokine receptors in addition to c-Kit (Figure 1). Mooney and colleagues observed that 13% of LT-HSC and 20% of ST-HSC expressed mRNA for the receptor for erythropoietin (EpoR), as examined by the qRT-PCR assay [15]. Whilst the lack of an antibody precluded the analysis of EpoR protein at the cell surface, early studies showed expression of EpoR protein at the surface of human HSCs, as measured using biotinylated recombinant Epo followed by a streptavidin conjugate [17]. Murine fetal B-cell/myeloid HPCs and adult HSCs express the receptor for macrophage colony-stimulating factor (M-CSFR) [18,19]. Nineteen percent of adult ST-HSC and 23% of ST-HSC express the M-CSFR at their cell surface, though very few of these cells (1% and 2%, respectively) expressed mRNA for the M-CSFR [15]. Other researchers have also reported that very few HSCs express this mRNA [19], and perhaps cells rarely transcribe the gene. Five percent of LT-HSCs and 8% of ST-HSCs express Flt3 at their cell surface.

Tpo chiefly regulates the production of megakaryocytes as this cytokine acts as a meagakaryocyte-colony stimulating and maturation factor. In addition to Tpo having this lineage specific role, murine HSCs (LSK CD34-ve Flt3-ve) express a high level of mRNA encoding the Tpo receptor (TpoR). From studies that used a chimeric construct encoding the extracellular domain of the TpoR and the cytoplasmic domain of the receptor for G-CSF to replace the TpoR gene, Stoffel and colleague argued that Tpo and G-CSF have a permissive role in HSC fate decision-making [20]. TpoR expression is downregulated in the more mature LSK cell population, that is CD34+ve and Flt3+ve [21], and investigators have proposed two opposing roles for Tpo: that are to expand HSCs during times of crisis and to maintain HSC quiescence [22]. LT-HSCs closely associate with Tpo-producing osteoblasts in niches in the bone marrow and niche signals may balance the two roles [23].

Transplantable murine HSCs express the receptor for G-CSF because G-CSF, as used with cyclophosphamide, mobilises HSCs from the bone marrow and causes them to proliferate prior to mobilisation [24]. Kondo and colleagues have shown that primitive murine HSCs expressed the receptor for GM-CSF at low to moderate levels and this receptor is absent on lymphoid progenitors [25].

The level of expression of the lineage-affiliated cytokine receptors at the surface of LT-HSCs and ST-HSCs, as measured by antibody labelling and FACS, is very low. We might therefore view the expression of the receptors as ‘primed’. Despite the limitation of a low level of expression, co-expression of mRNAs encoding Flt3 and EpoR rarely occurred, and in only two out of 139 single ST-HSCs analysed. Flt3 and M-CSFR co-expression occurred at the surface of a very small but significant fraction of LT-HSCs (1%) and ST-HSCs (3%) [15,26]. Presently, we have a somewhat incomplete picture of the cytokines that are important to lineage determination and limited information about the HSC co-expression of the above receptors. However, all of the receptors are regulated differentially following transition of HSCs to HPCs, and, for example, myeloid/lymphoid progenitors express Flt3 whereas progenitors developing towards megakaryocytes and erythrocytes do not [15]. A finding that is highly germane to the instructive action of M-CSF and G-CSF, particularly as ‘primed’ cells might have various options, is the fact that the expression of the receptors for these cytokines is positively autoregulated by cytokine binding. M-CSF binding to its receptor leads to expression of the transcription factor PU.1 that, in turn, upregulates M-CSFR expression. G-CSF receptor signalling increases the expression of the transcription factor C/EBPα that upregulates expression of the G-CSFR and that of the GM-CSFR (as reviewed in [8]). It is therefore possible that the presence of cytokine upregulates expression of the M-CSFR and the G-CSFR by LT-HSCs and ST-HSCs.

Commensurate with the lineage-affiliation of HSCs by virtue of cytokine receptor expression is that various approaches have revealed platelet-biased HSCs. The CD41 surface antigen (alpha11b intergrin, platelet GPIIb) is largely restricted to megakaryocytes, and Yamamoto and colleagues identified murine HSC that express CD41 at their cell surface that are repopulation-competent. They concluded that megakaryocyte lineage commitment occurs at the level of HSCs through a distinct pathway [27]. Murine HSCs that express a high level of c-Kit at their cell surface exhibited an intrinsic megakaryocytic lineage bias. As to their colony-forming ability in vitro, they produced higher frequencies of precursors with megakaryocyte potential, including committed megakaryocyte and pre-megakaryocyte/erythroid progenitors. When transplanted into mice, the c-Kit^high^ HSCs produced more megakaryocytes and platelets than c-Kit^low^ HSCs [28]. Around 60% of murine LT-HSC (LSK CD150+ve CD48-ve CD34-ve) express the megakaryocyte-affiliated von Willebrand factor and give rise to platelet- or platelet/myeloid-biased reconstitution when transplanted as single cells into irradiated mice [29].

## 3. The Instructive Action of Hematopoietic Cytokines

Recent findings have revealed that Epo, M-CSF, G-CSF and GM-CSF are instructive (Figure 1) and induce a signalling cascade and/or activate a genetic programme to commit HSCs and/or HPCs to adopting just one cell lineage. For these studies, investigators used HSCs and multi- and bipotent HPCs. There is still the need for more information from studies of ‘true’ HSCs, and regarding whether the instructive cytokines are able divert the end-cell option of biased HSCs (see above). Whilst the role of hematopoietic cytokines in determining a cell’s lineage remains controversial, there is good evidence to support an instructive role.

Grover and colleagues showed that Epo guides HSCs and multipotent HPCs towards an erythroid fate [30]. The investigators used a CMV-based Epo expression vector to increase the systemic level of Epo in mice to a level comparable to that observed in anemic patients. They plated bone marrow Lin-ve Sca1-ve c-Kit+ve cells, which contained myeloid/erythroid progenitors, in conditions to allow both myeloid and erythroid differentiation, and there was an increase in the proportion of erythroid colonies. LSK Flt3-ve cells belong to the HSCs/multipotent HPCs compartment and gene profiling of these cells post-exposure to Epo in vivo showed that erythroid commitment-associated genes were upregulated and megakaryocyte commitment- and granulocyte/macrophage commitment-associated genes were downregulation and there was no change to the size of the cell compartment. Epo had therefore altered the lineage programme of HSCs/multipotent HPCs. Treating LSK CD150+ve Flt3-ve cells with Epo in vitro led to the same alteration to the pattern of gene regulation. In support of skewing of the lineage potentials of these cells is the fact that transplantation of Epo-exposed HSCs/multipotent HPCs into sublethally irradiated mice generated higher numbers of erythrocytes and fewer myeloid cells. The overall findings are commensurate with an instructive effect of Epo that Gover and colleagues described as an “erythroid superhighway” stretching from HSCs via multipotent HPCs to the erythroid compartment.

As early as 1982, Metcalf and Burgess showed that M-CSF and GM-CSF respectively instruct macrophage and granulocyte fates within granulocyte/macrophage progenitor cells. In their experiments to reveal instruction they cultured each of the daughter cells of mouse granulocye-macrophage colony forming cells in either M-CSF or GM-CSF [31]. Until quite recently, it seems that the field paid little attention to the demonstration, by means of a clonal analysis, that M-CSF and GM-CSF are instructive, at least for bipotent progenitors. In 2009, Reiger and colleagues provided more evidence to support the view that M-CSF instructs mouse granulocyte/macrophage progenitors to adopt a macrophage fate and showed that G-CSF instructs granulocyte fate [32]. They used bioimaging approaches to follow granulocyte-macrophage progenitor cells and the development of all their progeny into macrophage- or granulocyte-committed cells when cultured in the presence of only M-CSF or G-SCF. Reiger and colleagues observed that M- and G-CSF instructed at least 65% and 34% of bipotent cells to differentiate into the M and G lineage, respectively. As above, HSCs can choose a fate and within the cell population studied there were cells that were already committed to a fate, estimated at 23% and 53% macrophage and granulocyte unilineage-restricted progenitors, respectively. To extend the instructive action of M-CSF to HSCs, Mossadegh-Keller and colleagues showed that M-CSF directly induces the master myeloid regulator PU.1 in single HSCs, that in turn, instructs myeloid lineage fate. Single-cell gene video imaging and expression analysis confirmed activation of the PU.1 promoter and an increased number of PU.1+ve cells with a myeloid signature post-treatment of highly purified HSCs with M-CSF in culture. Here, we see a direct action of a cytokine instructing a change of identity [19]. As revealed from studies of Flt3L transgenic mice, the instructive action of Flt3L diverts murine LSK cells towards myeloid/lymphoid development and suppresses the generation of megakaryocyte and erythroid progenitors. Very high levels of exposure of mice to Flt3L also led to the development of anemia together with reduced platelet numbers. A rapid drop in the number of the earliest erythroid progenitors was in keeping with a direct instructive and negative role on this pathway [33]. As above, the level of Epo used to instruct lineage fate was also high.

## 4. HSC Commitment to Differentiation and Finding a New Niche

Some hematopoietic cytokines act on HSCs and HPCs in a permissive manner to control survival and proliferation and in an instructive manner to govern lineage fate. As illustrated by the action of Flt3 ligand, the concentration of the cytokine and therefore the strength of signal received by the cell are important. The level of FLt3 ligand that was required to drive cells towards a lymphoid/myeloid fate substantially exceeded the level experienced by cells under steady-state conditions [33]. However and under stress and emergency conditions, for example inflammation, infection, bleeding or injury, cytokine levels do increase dramatically, which may divert developing cells towards a particular lineage to increase the production of an end cell type.

Concentration is important because, in addition to pro-survival, pro-proliferation and lineage instructive actions, some hematopoietic cytokines are chemotactic factors whereby a gradient is paramount. SCF is a chemoattractant for HPCs [6]. HPCs are able to sense a gradient of SCF as revealed from studies by Colmore and colleagues using a severe combined immunodeficiency mouse xenograft model of human HPCs (CD34+ve cells from cord and peripheral blood) and Nalm-6 pre-B acute lymphoblastic leukemia cells. These leukemia cells created abnormal bone marrow niches and the human HPCs honed into the malignant vascular niches. The malignant niches also competed for HPCs previously established in normal bone marrow niches. SCF secreted by the engrafted Nalm-6 cells was responsible for the cell migration towards the malignant niches and the sequestering HPCs [34].

Like HSCs, HPCs reside in particular niches where adjacent stromal and mesenchymal cells provide maintenance factors and factors that can change the behaviour of resident cells. Investigators described the niches for erythropoiesis, termed erythroblastic islands, more than 50 years ago from the analysis of transmission electron micrographs of bone marrow [35]. These niches are specialised, providing Epo to complete an autonomous and terminal-erythroid differentiation program. Additionally, restricted HPCs and erythropoiesis requires SCF from bone marrow stromal cells that are leptin receptor positive, whereas HSCs require SCF from endothelial cells. In keeping, erythroid progenitors localise adjacent to perisinusoidal leptin receptor positive cells [36]. Perhaps HSCs that are primed by virtue of expression of the EpoR, and destined for erythropoiesis, need to find an appropriate niche. We know that Epo is a chemoattractant that increases the migration of mesenchymal stem cells [37], endothelial cells [38] and human neuroblastoma cells [39]. Examination of whether Epo can also guide the migration of EpoR+ve HSCs/HPCs within bone marrow would be technically very demanding and therefore we can only speculate whether Epo has such a role.

GM-CSF is a powerful chemoattractant for cells bearing the receptor as shown by Gomez-Cambronero and colleagues for human neutrophils, peripheral blood monocytes and the HL60 promyeloid and myeloproliferative disorder (MPD) cell lines following induction of differentiation towards neutrophils. All these cells express the GM-CSFR, and peripheral blood lymphocytes that do not express GM-CSFR did not respond to the cytokine [40]. Other authors have shown that GM-CSF is a chemoattractant for endothelial [41] and mesenchymal cells [42]. GM-CSFR-bearing cells respond in a chemotactic manner to GM-CSF, and therefore we might expect GM-CSFR+ve HSCs and HPCs to do so. Similarly, M-CSF is chemotactic for cells that have M-CSFR including osteoclasts [43], monocytes [44], macrophages [45] and myeloid progenitor cells (32D) transfected with the M-CSFR [46]. M-CSF also influences the migration through tissues of trophoblastic cells [47] and that of some M-CSFR-expressing breast cancer cells [48]. From the findings from some quite early studies, we can add chemotaxis to the actions of GM-CSF and M-CSF, which might have an important bearing on the instructive roles of these cytokines.

## 5. Natural Variation within Hematopoietic Stem Cells

Quite simply, all HSCs, and other cells within a given population, are not the same, as seen for HSCs from surface marker analyses and different developmental propensities. A more detailed analysis of early progenitors with lymphoid and myeloid potential (EPLM) overturned efforts to ring-fence cells as a homogenous population by the use of a large panel of cell surface markers. Single cell sequencing provides a much higher resolution of different cell states than mapping by surface marker analysis, or by examining the ultimate fate of cells in vitro or in vivo. Alberti-Servera and colleagues examined the signatures of a primitive subpopulation of EPLM, lacking expression of the markers Ly6D, SiglecH and CD11c, by RNA sequencing of single cells and observed that they are really a mixture of cells with either myeloid, dendritic cell or lymphoid signatures [49]. Lineage tracing from the transcriptional landscapes of cells within DNA barcoded HPC clones has revealed lineage priming within early progenitors and that monocytes differentiate via multiple trajectories. Weinreb and colleagues used coloured outlines to circumscribe probable regions of fate progression with cell potentials located on a continuous transcriptional landscape, rather than drawing lines to prescribe preferred pathways [50]. From the analysis of mRNA expression data for single HSCs and HPCs, Nestora and colleagues also arrived at the conclusion that the trajectories of developing cells are broad and cells can veer towards an alternative pathway(s) [51]. A report that explained stem cell behaviour using a computer stimulation supports this point of view [52].

There is therefore natural variation within HSCs that is inevitable because the chemical reactions within a cell are random in their nature. There is inherent random noise/variation in the level of expression of genes [53] and an inherent dynamic variation in the DNA methylation patterns across DNA and/or activating and repressive histones modifications that engrave the status of chromatin [54]. As to the underlying importance of the chromatin structure, noise in the distribution of levels of mRNA (known as translational noise) within a population of cells intriguingly correlates with the three-dimensional organisation of nuclear domains [55]. SATB1 is involved in the three-dimensional organization of nuclear domains and variable levels regulate HSC heterogeneity regarding distinct lineage fates [56]. In 2008, Chang and colleagues brought to attention the fact that the cell-to-cell variability of clonal populations of mouse HPCs is a manifestation of ‘gene expression noise’. They concluded that transcriptome-wide noise controls lineage choice in HPCs [57]. Moreover, autonomous fluctuations seem to prime these cells before they receive environmental signals.

The above events most likely underpin cell variation and the differences extend to the expression of cytokine receptors, providing a means for cells to adapt their behaviour. The ‘success’ of a cell in contributing to an organism as a whole might well relate to its inherent capacity to adapt to another environment to keep pace with changing environmental conditions. Coexpression of the receptor for SCF and a lineage-affiliated receptor(s) should provide a survival advantage to HSCs and HPCs if, for example, the supply of SCF becomes scare. Adaptability might also include the rewiring of the lineage fate of HSCs and HPCs to guide them towards a new identity so that the supply of different cell types meets the overall societal requirements of an organism.

## 6. A Natural Selection Model of Hematopoiesis

**Figure 2:**
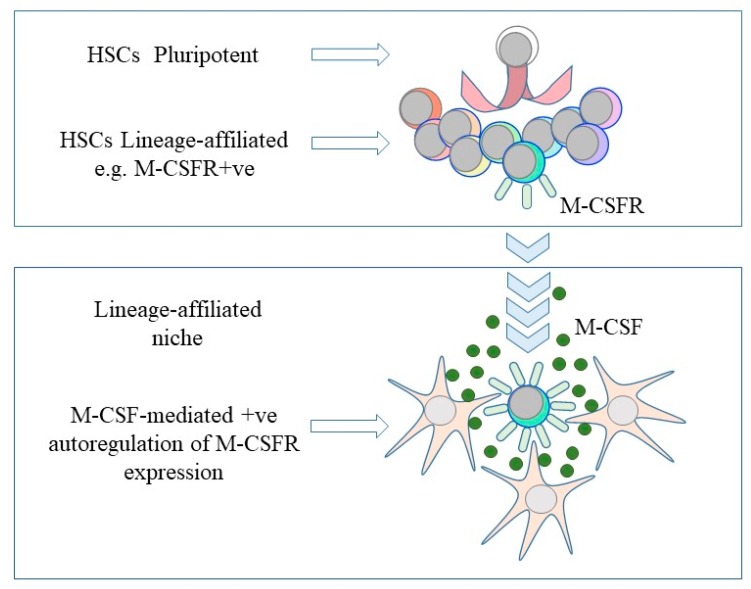
A continuum and niche model of the fate of hematopoietic stem cells. Hematopoietic stem cells (HSC) choose directly from all of the available fates and are primed towards a cell lineage by virtue of a low level of expression of the receptor for a lineage-affiliated cytokine, for example, the receptor for macrophage colony-stimulating factor (M-CSFR). The provision of, for example, macrophage colony-stimulating factor (M-CSF), bound to proximal cells and/or the extracellular matrix, positively autoregulates expression of the receptor to enforce development along the ‘chosen’ pathway.

Findings, both recent and longstanding, that lead towards a new understanding of the fates of HSCs are that some are affiliated to just one cell lineage, some hematopoietic cytokines instruct lineage fate and the environment HSCs and HPCs reside in supports their development. From these three premises, we might propose a natural selection/Darwinist model of hematopoiesis (Figure 2). In the first instance, HSCs chooses directly, whether randomly or in an ordered manner, from all of the available fates and are primed towards a cell lineage(s) by virtue of a low level of expression of the receptor for a lineage-affiliated cytokine(s), for example, M-CSF. The then lineage-affiliated/biased cell undergoes development by ‘finding’ an appropriate niche to do so. The niche might attract the cell via the chemoattractant action of a cytokine(s)—other than for SCF and HSCs this is largely untested. The provision of the lineage-affiliated cytokine within the niche, for example M-CSF, then positively autoregulates expression of the receptor, and in doing so enforces development along the ‘chosen’ pathway. However, Ceredig and I have argued that HSCs and HPCs are versatile and retain the ability to move along a pathway that is different from the one chosen initially [8]. Regarding the dynamism of these cells, a recent review agrees with this notion presenting a view of hematopoietic cell biology from different viewpoints, including dynamic physics and the social network theory [58]. If the niches enforce the fate of a cell, they might accommodate versatility by means of a capacity to present various cytokines pending their availability. In this scenario, the output of the balance of the mature cell types generated is a combination of HSCs veering first towards just one pathway, though still able to ‘change their mind’, and the mosaic within the bone marrow of the different cytokine-enriched niches that enforce and support developmental progression.

That bone marrow niches are important the survival and proliferation of HSCs and HPCs in a permissive manner is a longstanding principle. We now view some cytokines as instructive, and it is highly likely that stromal, epithelial or mesenchymal cells present them to developing cells rather than that they are soluble molecules. That environment domains/niches are zones that govern lineage fate is by no means original. A precedent is from the elegant studies of how plant cells make a fate choice, and most of the knowledge comes from studies of *Arabidopsis*. Plants have stem cells, their three-dimensional cell patterning is simple and clear and the dominance of position-dependent cell-fate commitment is widely accepted. The later leaves of *Arabidopsis* have epidermal hairs (trichomes) just at the tip, and a wave that forms at the tip and that moves towards the base of the developing leaf defines the pattern, and the timing of, epidermal hair formation. Each epidermal leaf cell has the probability of differentiating into epidermal hairs and a steroid-inducible regulator defines a zone of cell-fate decision-making. Individual cells enter a zone from the base and exit towards the tip of the leaf, and they can no longer adopt a trichome fate once they have left the zone in which decision-making occurs [59]. Similarly, conversion of cells from an epidermis to a cortex fate occurs in the roots of *Arabidopsis* with positional information reprograming the initial cell identity [60]. Abandonment of a zonal aspect to cell fate decision-making by mammals seems unlikely, and, of course, we know that HSCs residence in a particular niche is very important to the choice between self-renewal versus differentiation.

## 7. Concluding Remarks

Since the 1980s, describing the conduct of hematopoiesis focused somewhat on drawing treelike maps to prescribe the routes that the progeny of pluripotent HSCs invariably follow to give rise to each type of end-cell. There are many different maps showing the stepwise progression of HSCs via intermediate oligo-potent and ultimately bipotent progenitors. Perhaps the need to put lines on a diagram of the architecture of hematopoiesis no longer exists because we now know that HSCs are a heterogeneous population of cells with some cells affiliated directly to just one cell lineage [7,8]. Even when they have ‘chosen’ a pathway, HSCs and HPCs can still change their mind to adopt another lineage. There are ‘influences’ on cell fate that are not detectable by single-cell RNA sequencing, and the environment a cell resides in, that in turn, modulates the epigenetic landscape [61], has a prominent role in cell fate decision-making. For some hematopoietic cytokines, there appears to be a resolution to the debate about a permissive (for survival and proliferation) versus an instructive (for lineage fate) action because new findings have shown that some cytokines have both roles. Lineage-affiliated HSCs need to expand to replenish mature cells and perhaps a low receptor signal-strength provokes their proliferation and a much high signal-strength is required to guarantee diversion towards a pathway, as seen for Flt3L and Epo. Some of the instructive cytokines are also chemoattractants. Bone marrow is a solid and dynamic tissue and therefore cell migration is important. The environment that cells migrate through is complex, to the extent that cells can even convert mechanical cues, such as stiffness, into biochemical signals that affect the ability to self-renew, differentiate and ensure cell-type commitment [62]. We still do not know how HSCs selectively express lineage-affiliated cytokine receptors in the first instance, nor whether individual cells are able to prime a range of fate options simultaneously or do so one at a time. In summary, providing an understanding of the process of HSC decision-making is much more complex than drawing treelike maps to dictate pathways.

## References

[1] Schofield R. (1978). The relationship between the spleen colony-forming cell and the haematopoietic stem cell. Blood Cells.

[2] Pinho S., Frenette P.S. (2019). Haematopoietic stem cell activity and interactions with the niche. Nat. Rev. Mol. Cell Biol..

[3] Okumura N., Ebihara Y., Tanaka I., Koike K., Komiyama A., Nakahata T. (1996). Chemotactic and chemokinetic activities of stem cell factor on murine hematopoietic progenitor cells. Blood.

[4] Driessen R.L., Johnson H.M., Nilsson S.K. (2003). Membrane-bound stem cell factor is a key regulator in the initial lodgement of stem cells within the endosteal marrow region. Exp. Hematol..

[5] Ashman L.K. (1999). The biology of stem cell factor and its receptor C-kit. Int. J. Biochem. Cell. Biol..

[6] Sharma S., Gurudutta G.U., Satija N.K., Pati S., Gupta P., Verma Y.K., Singh V.K., Tripathi R.P. (2006). Stem cell c-KIT and HOXB4 genes: Critical roles and mechanisms in self-renewal, proliferation, and differentiation. Stem Cells Dev..

[7] Ceredig R., Rolink A.G., Brown G. (2009). Models of haematopoiesis: Seeing the wood for the trees. Nat. Rev. Immunol..

[8] Brown G., Ceredig R. (2019). Modelling the hematopoietic landscape. Front. Cell Dev. Biol..

[9] Hume D.A., MacDonald K.P. (2012). Therapeutic applications of macrophage colony-stimulating factor (CSF-1) and antagonists of CSF-1 receptor (CSF-1R) signaling. Blood.

[10] Koury M.J., Bondurant M.C. (1990). Erythropoietin retards DNA breakdown and prevents programmed death in erythroid progenitor cells. Science.

[11] Demetri G.D., Griffin J.D. (1991). Granulocyte colony-stimulating factor and its receptor. Blood.

[12] Metcalf D. (1993). Hematopoietic regulators: Redundancy or subtlety?. Blood.

[13] Gasson J.C. (1991). Molecular physiology of granulocyte-macrophage colony stimulating factor. Blood.

[14] Kaushansky K. (1995). Thrombopoietin: The primary regulator of megakaryocyte and platelet production. Thromb. Haemost..

[15] Mooney C.J., Cunningham A., Tsapogas P., Toellner K.-M., Brown G. (2017). Selective expression of Flt3 within the mouse hematopoietic stem cell compartment. Int. J. Mol. Sci..

[16] Osawa M., Hanada K., Hamada H., Nakauchi H. (1996). Long-term lymphohematopoietic reconstitution by a single CD34-low/negative hematopoietic stem cell. Science.

[17] Shinjo K., Takeshita A., Higuchi M., Ohnishi K., Ohno R. (1997). Erythropoietin receptor expression on human bone marrow erythroid precursor cells by a newly-devised quantitative flow-cytometric assay. Br. J. Haematol..

[18] Zriwil A., Bolers C., Wittmann L., Green J.C.A., Woll P.S., Jacobsen S.E.W., Sitnicka E. (2016). Macrophage colony-stimulating factor receptor marks and regulates a fetal myeloid-primed B-cell progenitor in mice. Blood.

[19] Mossadegh-Keller N., Sarrazin S., Kandalla P.K., Espinosa L., Stanley E.R., Nutt S.L., Moore J., Sieweke M.H. (2013). M-CSF instructs myeloid lineage fate in single haematopoietic stem cells. Nature.

[20] Stoffel R., Ziegler S., Ghilhardi N., Ledermann B., de Sauvage F.J., Skoda R.C. (1999). Permissive role of thrombopoietin and granulocyte colony-stimulating factor receptors in hematopoietic cell fate decisions in vivo. Proc. Natl. Acad Sci. USA.

[21] Mansson R., Hultquist A., Luc S., Yang L., Anderson K., Kharazi S., Al-Hashmi S., Liuba K., Thoren L., Adolffsson J. (2007). Molecular evidence for hierarchical transcriptional lineage priming in fetal and adult stem cells and multipotent progenitors. Immunity.

[22] De Graaf C., Metcalf D. (2011). Thrombopoietin and hematopoietic stem cells. Cell Cycle.

[23] Yoshihara H., Arai F., Hosokawa K., Hagiwara T., Takubo K., Nakamura Y., Gomei Y., Iwasaki H., Matsuoka S., Miyanoto K. (2007). Thrombopoietin/MPL signaling regulates hematopoietic stem cell quiescence and interaction with the osteoblastic niche. Cell Stem Cell.

[24] Morrison S.J., Wright D.E., Weissman I.L. (1997). Cyclophosphamide/granulocyte colony-stimulating factor induces hematopoietic stem cells to proliferate prior to mobilization. Proc. Natl. Acad. Sci. USA.

[25] Kondo M., Scherer D.C., Miyamoto T., King A.G., Akashi K., Sugamura K., Weissman I.L. (2000). Cell-fate conversion of lymphoid-committed progenitors by instructive actions of cytokines. Nature.

[26] Tsapogas P., Mooney C.J., Brown G., Rolink A. (2017). The cytokine Flt3-ligand in normal and malignant hematopoiesis. Int. J. Mol. Sci..

[27] Yamamoto R., Morita Y., Ooehara J., Hamanaka S., Onodera M., Rudolph K.L., Ema H., Nakauchi H. (2013). Clonal analysis unveils self-renewing lineage-restricted progenitors generated directly from hematopoietic stem cells. Cell.

[28] Shin J.Y., Hu W., Naramura M., Park C.Y. (2014). High c-Kit expression identifies hematopoietic stem cells with impaired self-renewal and megakaryocytic bias. J. Exp. Med..

[29] Sanjuan-Pla A., Macaulay I.C., Jensen C.T., Woll P.S., Luis T.C., Mead A., Moore S., Carella C., Bouriez Jones T., Chowdhury O. (2013). Platelet-biased stem cells reside at the apex of the hematopoietic stem-cell hierarchy. Nature.

[30] Grover A., Mancini E., Moore S., Mead A.J., Atkinson D., Rasmussen K.D., O’Carroll D., Jacobsen S.E., Nerlove C. (2014). Erythropoietin guides multipotent hematopoietic progenitor cells towards an erythroid fate. J. Exp. Med..

[31] Metcalf D., Burgess A.W. (1982). Clonal analysis of progenitor cell commitment of granulocyte or macrophage production. J. Cell Physiol..

[32] Rieger M.A., Hoppe P.S., Smejkal B.M., Eitelhuber A.C., Schroeder T. (2009). Hematopoietic cytokines can instruct lineage choice. Science.

[33] Tsapogas P., Swee L.K., Nusser A., Nuber N., Kreuzaler M., Capoferri G., Rolink H., Ceredig R., Rolink A. (2014). In vivo evidence for an instructive role of fms-like tyrosine kinase-3 (FLT3) ligand in hematopoietic development. Haematologica.

[34] Colmore A., Amorim M., Pontier A.L., Wang S., Jablonski E., Sipkins D.A. (2008). Leukaemia cells create bone marrow niches that disrupt the behaviour of normal hematopoietic progenitor cells. Science.

[35] Chasis J.A., Mohandas N. (2008). Erythroblastic islands: Niches for erythropoiesis. Blood.

[36] Comazzetto S., Murphy M.M., Berto S., Jeffery E., Zhao Z., Morrison S.J. (2019). Restricted hematopoietic progenitors and reythropoiesis require SCF from leptin receptor+ niche cells in bone marrow. Cell Stem Cell.

[37] Zwezdaryk K.J., Coffelt S.B., Figueroa Y.G., Liu J., Phinney D.G., LaMarca H.L., Florez L., Morris C.B., Hoyle G.W., Scandurro A.B. (2007). Erythropoietin, a hypoxia-regulated factor, elicits a pro-angiogenic program in human mesenchymal stem cells. Exp. Hemato..

[38] Anagnostou A., Lee E.S., Keissiman N., Levinson R., Steiner M. (1990). Erytrhopoietin has a mitogenic and postive chemotactic effect on endothelial cells. Proc. Natl. Acad. Sci. USA.

[39] Poniewierska-Baran A., Rajewska J.R., Ratajczak M.Z. (2017). Erythropoietin enhances migration of human nuroblastoma cells: In vitro studies and potential therapeutic implication. J. Cancer Stem Cell Res..

[40] Gomez-Cambronero J., Horn J., Paul C.C., Baumann M.A. (2003). Granulocyte-macrophage colony-stimulating factor is a chemoattractant cytokine for human neutrophils: Involvement of the ribosomal p70 S6 kinase signalling pathway. J. Immunol..

[41] Bussolino F., Wang J.M., Defilippi P., Turrini F., Sanavio F., Edgell J., Aglietta M., Arese P., Mantovani A. (1989). Granulocyte- and granulocyte-macrophage-colony stimulating factors induce human endothelial cells to migrateand proliferate. Nature.

[42] Vaillant P., Muller V., Martinet Y., Martinet N. (1993). Human granulocyte and granulocyte-macrophage-colony stimulating factors are chemotactic and “competence” growth factors for human mesenchymal cells. Biochem. Biophys. Res. Commun..

[43] Fuller K., Owens J.M., Jagger A., Wilson A., Moss R., Chambers T.J. (1993). Macrophage colony-stimulating factors stimulates survival and chemotactic behaviour in isolated osteoclasts. J. Exp. Med..

[44] Wang J.M., Griffin J.D., Rambaldi A., Chen Z.D., Mantovani A. (1988). Induction of monocyte migration by recombinant macrophage colony-stimulating factor. J. Immunol..

[45] Vedham V., Phee H., Coggeshall K.M. (2005). Vav activation and function as a Rac guanine nucleotide exchange factor in macrophage colony-stimulating factor-induced macrophage chemotaxis. Mol Cell Biol..

[46] Pierce J.H., Di Marco E., Cox G.W., Lombardi D., Ruggiero M., Varesio L., Wang L.M., Choudhury G.C., Sakaguchi A.Y., Di Fiore P.P. (1990). Macrophage-colony-stimulating factor (CSF-1) induces proliferation, chemotaxis, and reversible monocytic differentiation in myeloid progenitor cells transfected with human c-fms/CSF-1 receptor cDNA. Proc. Natl. Acad. Sci. USA.

[47] Pollard J.W., Bartocci A., Arceci R., Orlofsky A., Ladner M.B., Stanley E.R. (1987). Apparent role of the macrophage growth factor, CSF-1, in placental development. Nature.

[48] Filderman A.E., Bruckner A., Kacinski B.M., Deng N., Remold H.G. (1992). Macrophage colony-stimulating factor (CSF-1) enhances invasiveness in CSF-1 receptor-positive carcinoma cell lines. Cancer Res..

[49] Alberti-Servera L., von Muenchow L., Tsapogas P., Capoferri G., Eschbach K., Beisel C. (2017). Single-cell RNA sequencing reveals developmental heterogeneity among early lymphoid progenitors. EMBO J..

[50] Weinreb C., Rodriguez-Fraticelli A.R., Camargo F., Klein A.M. (2020). Lineage tracing on transcriptional landscapes links state to fate during differentiation. Science.

[51] Nestorowa S., Hamey F.K., Pijuan Sala B., Diamanti E., Shepherd M., Laurenti E., Wilson N.K., Kent D.G., Gottens B. (2016). A single-cell resolution map of mouse hematopoietic stem and progenitor cell differentiation. Blood.

[52] Furusawa C., Kaneko K. (2012). A dynamical-systems view of stem cell biology. Science.

[53] Raser J.M., O’Shea E.K. (2005). Noise in gene expression: Origins, consequences, and control. Science.

[54] Lund R.J., Narva E., Lahesmaa R. (2012). Genetic and epigenetic stability of human pluripotent stem cells. Nat. Rev. Genet..

[55] Barroso G.V., Puzovic N., Dutheil J.Y. (2018). The evolution of gene-specific transcriptional noise is driven by selection at the pathway level. Genetics.

[56] Doi Y., Yokota Y., Satoh Y., Okuzaki D., Tokunaga M., Ishibashi T., Sudo T., Ueda T., Shingai Y., Ichi M. (2018). Variable SATB1 levels regulate hematopoietic stem cell heterogeneity with distinct lineage fate. Cell Rep..

[57] Chang H.H., Hemberg M., Barahana M., Ingber D.E., Huang S. (2008). Transcriptome-wide noise controls lineage choice in mammalian progenitor cells. Nature.

[58] Yokota T. (2019). “Hierarchy” and “Holoaciary”: A paradigm of the hematopoietic system. Cells.

[59] Lloyd A.M., Schena M., Walbot V., Davis R.W. (1994). Epidermal cell fate determination in Arabidopsis: Patterns defined by a steroid-inducible regulator. Science.

[60] Yu Q., Li P., Liang N., Wang H., Xu M., Wu S. (2017). Cell-fate specification in Arabidopsis roots requires coordinative action of lineage instruction and positional reprogramming. Plant Physiol..

[61] Szyf M., McGowan P.M., Meaney M.J. (2008). The social environment and the epigenome. Environ. Mol. Mutagenesis.

[62] Bloom A.B., Zaman M.H. (2014). Influence of the microenvironment on cell fate determination and migration. Physiol. Genomics.

